# Impact of a Diagnosis-Centered Antibiotic Stewardship on Incident *Clostridioides difficile* Infections in Older Inpatients: An Observational Study

**DOI:** 10.3390/antibiotics9060303

**Published:** 2020-06-05

**Authors:** Alain Putot, Karine Astruc, Jeremy Barben, Anca Maria Mihai, Valentine Nuss, Julien Bador, Sophie Putot, Mélanie Dipanda, Caroline Laborde, Jeremie Vovelle, Sofia Da Silva, Emmanuel Mazen, Ludwig Serge Aho Glélé, Patrick Manckoundia

**Affiliations:** 1 Service de Médecine Interne Gériatrie, Hôpital de Champmaillot, Centre Hospitalier Universitaire, 2 rue Jules Violle, 21079 Dijon CEDEX, France; jeremy.barben@chu-dijon.fr (J.B.); anca.mi17@yahoo.com (A.M.M.); valentine.nuss@chu-dijon.fr (V.N.); sophie.putot@chu-dijon.fr (S.P.); melanie.dipanda@chu-dijon.fr (M.D.); caroline.laborde@chu-dijon.fr (C.L.); Jeremie.vovelle@chu-dijon.fr (J.V.); sophia.dasilva@chu-dijon.fr (S.D.S.); emmanuel.mazen@chu-dijon.fr (E.M.); patrick.manckoundia@chu-dijon.fr (P.M.); 2 Service d’Epidémiologie et Hygiène Hospitalière, Centre Hospitalier Universitaire, 14 rue Paul Gaffarel, 21079 Dijon CEDEX, France; karine.astruc@chu-dijon.fr (K.A.); ludwig.aho@chu-dijon.fr (L.S.A.G.); 3 Service de Microbiologie, Centre Hospitalier Universitaire, 14 rue Paul Gaffarel, 21079 Dijon CEDEX, France; julien.bador@chu-dijon.fr

**Keywords:** Clostridioides difficile, clostridium, stewardship, diagnostic, acute infection, antibiotic, aged

## Abstract

In 2015, a major increase in incident hospital-onset Clostridioides difficile infections (HO-CDI) in a geriatric university hospital led to the implementation of a diagnosis-centered antibiotic stewardship program (ASP). We aimed to evaluate the impact of the ASP on antibiotic consumption and on HO-CDI incidence. The intervention was the arrival of a full-time infectiologist in the acute geriatric unit in May 2015, followed by the implementation of new diagnostic procedures for infections associated with an antibiotic withdrawal policy. Between 2015 and 2018, the ASP was associated with a major reduction in diagnoses for inpatients (23% to 13% for pneumonia, 24% to 13% for urinary tract infection), while median hospital stays and mortality rates remained stable. The reduction in diagnosed bacterial infections was associated with a 45% decrease in antibiotic consumption in the acute geriatric unit. HO-CDI incidence also decreased dramatically from 1.4‰ bed-days to 0.8‰ bed-days in the geriatric rehabilitation unit. The ASP focused on reducing the overdiagnosis of bacterial infections in the acute geriatric unit was successfully associated with both a reduction in antibiotic use and a clear reduction in the incidence of HO-CDI in the geriatric rehabilitation unit.

## 1. Introduction

*Clostridioides difficile* is frequently responsible for healthcare-associated infections in the older hospitalized population. Hospital-onset *Clostridioides difficile* infections (HO-CDI) place a major burden on acute and post-acute geriatric units, in both the short and long terms [1]. One particularly important risk factor at the patient-level for HO-CDI is previous exposure to antibiotics. However, in hospitals, incident HO-CDI has also been shown to be correlated with antibiotic consumption [2], and reductions in antibiotic use have been associated with lower HO-CDI incidence [3,4,5,6,7,8]. Antimicrobial stewardship programs (ASP) have proven efficient in decreasing CDI incidence in geriatric units [3,9]. 

In 2015, our university hospital faced an outbreak of HO-CDI in the acute geriatric unit (AGU) and in the geriatric rehabilitation unit (GRU) without an identified cluster, while HO-CDI incidence remained stable in the rest of the hospital. Considering that HO-CDI incidence is linked to antibiotic consumption, an ASP was established in the AGU to optimize the prescription of antimicrobial drugs. Assuming that most of the opportunities to reduce HO-CDI were linked to unnecessary antimicrobials [10], this ASP especially focused on redressing misdiagnosis of bacterial infection at the patient’s bedside. 

We aimed to evaluate the efficiency of this procedure on the antibiotic consumption in the AGU and on HO-CDI incidence in the GRU following acute care.

## 2. Methods

### 2.1. Patients

All patients hospitalized in the AGU (62 beds) and the GRU (68 beds) of our university hospital from January 2013 to December 2018 were retrospectively included in this observational study. These geriatric units are specialized in the management of multimorbid patients aged 75 and over. About 80% of patients hospitalized in GRU arrive from the AGU after the acute phase of illness. 

Diagnoses of HO-CDI, pneumonia, urinary tract infections (UTI), and viral respiratory tract infection (RTI) were obtained using the national regulatory procedure coding system from the French medical information system program (PMSI) and expressed in percentage of inpatients. There was no change in coding procedure during this period. There was also no change in HO-CDI diagnosis procedure into our hospital, in accordance with European guidelines [11]. All the HO-CDI patients were isolated according to the current recommendations for CDI complementary hygiene measures during the whole study period [12].

The yearly quantities of antibiotics delivered to AGU and GRU were obtained from the hospital pharmacy information system and were converted into defined daily doses (DDDs) per 1000 bed-days (BD) following the recommendations of the World Health Organization (WHO) [13].

The in-hospital mortality rate per year was also obtained from the PMSI database.

### 2.2. Antibiotics Stewardship Program

The following procedures were implemented in the AGU on the recommendations of a senior infectiologist specialized in older patients, who became a full-time staff member in this unit in May 2015:

Immediate discontinuation of antibiotic therapy in the absence of sepsis or clinical evidence of bacterial infection, especially discontinuation of antimicrobial treatment introduced for the following three very frequent situations: (1) isolated inflammatory syndrome, (2) positive urine dipstick or cytobacterial examination without signs or symptoms [14], and (3) acute respiratory symptoms without respiratory distress or positive diagnosis of acute pneumonia.

In addition, the AGU implemented the following diagnostic strategies that have been shown to decrease antibiotic consumption:

Systematic documentation of acute respiratory symptoms in a viral epidemic context with multiplex viral real-time PCR (Argene, Varilhes, France) [15] to avoid or stop unnecessary antibiotic therapy in documented cases of viral RTI.

Use of procalcitonin [16] and chest tomodensitometry [17] in case of doubt for the diagnosis of pneumonia after clinical examination and conventional radiography.

In cases of acute respiratory failure, use of bed-side transthoracic ultrasound [18] by two senior geriatricians, in order to obtain a positive diagnosis of pneumonia or differential diagnoses (especially acute heart failure).

These recommendations were implemented through formal presentations to the medical staff and ongoing bedside expertise, provided by the infectiologist who has been named to the AGU.

## 3. Results

### 3.1. Infection Diagnoses and Antibiotics Consumption

**Figure 1:**
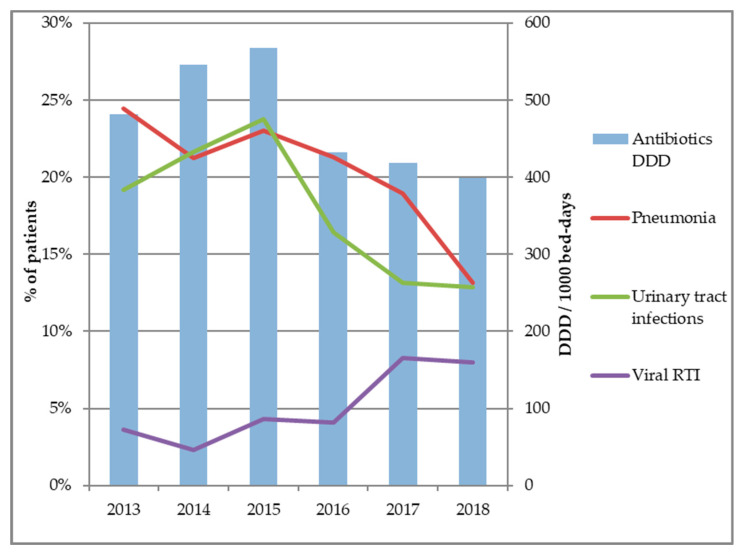
Frequency of main bacterial infection diagnoses and yearly antibiotic consumption in the acute geriatric unit. DDD: defined daily doses, RTI: respiratory tract infection.

During the first period of this study (2013–2015), antibiotic consumption in the AGU increased from 480 DDD to 550 DDD/1000 BD (Figure 1). After the implementation of the ASP (2015–2018), the antibiotic consumption dramatically decreased from 27%, from 550 DDD to 400 DDD/1000 BD.

This decrease of antibiotic consumption was concurrent with a major decrease in the diagnoses of incident acute pneumonia (23% to 13% of patients) and UTI (24% to 13% of patients), while viral RTI increased significantly during the same period (4% to 7% of patients). Median length of hospitalization (10 days) and in-hospital mortality (9%) in the AGU remained constant throughout the study.

### 3.2. Antibiotic Consumption and HO-CDI Incidence 

**Figure 2:**
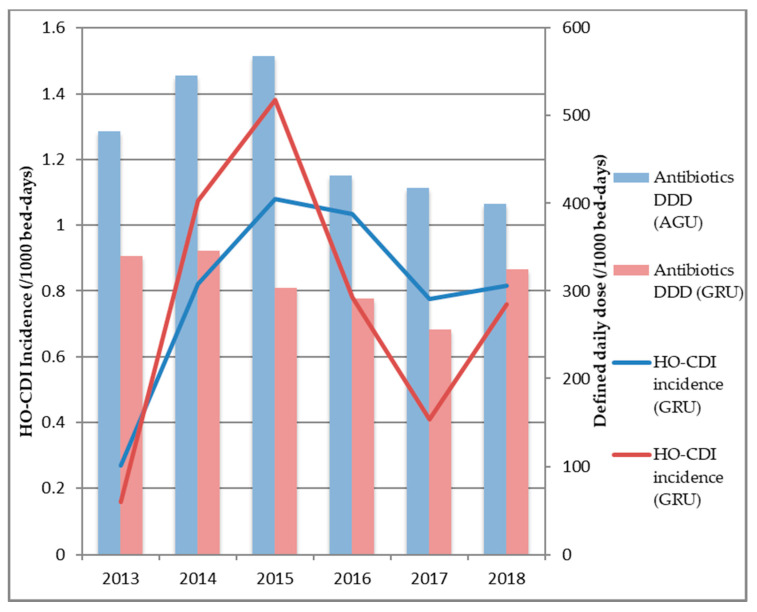
Antibiotic consumption and incidence of hospital onset *C. difficile* infection (HO-CDI) in acute geriatric unit (AGU) and geriatric rehabilitation unit (GRU).

While in the AGU, the antibiotic consumption decreased during the ASP period AGU, it remained roughly stable in the GRU (Figure 2). However, HO-CDI incidence in the GRU dramatically increased from 0.2/1000 BD in 2013 to 1.4/1000 BD in 2015, which justified the implementation of the ASP in May 2015. 

In the ASP period, concurrently with the decreased consumption of antibiotics in the AGU, the incidence of HO-CDI in the GRU dropped considerably to 0.8/1000 BD in 2018. Similar trends were observed in the AGU (Figure 2).

## 4. Discussion

Our study found that the ASP was associated with a reduction in HO-CDI incidence, which was consistent with previous reports in geriatric settings [3,9]. However, ASPs that target only high-cost, broad-spectrum antimicrobials can miss opportunities to reduce HO-CDI due to the overuse of unaudited low-cost and often unnecessary antimicrobials [10]. We chose not to focus our ASP on any specific antibiotic treatment, but aimed to reduce the overdiagnosis of bacterial infections. Antibiotic restriction is particularly difficult in a geriatric setting. Because the clinical presentation of infection is frequently atypical in the elderly, antibiotics are widely prescribed when the clinical presentation is aspecific. Many patients hospitalized with a diagnosis of bacterial infection are not considered ill with this infection at discharge [19]. However, several diagnostic tools presented in the methods, including biochemical, microbiological, and radiological data, have proven efficient for improving diagnostic sensitivity, especially to differentiate acute pneumonia from viral RTI or acute heart failure. 

In our study, the diagnosis-centered ASP implemented in the AGU was followed by a near half reduction in the diagnoses of bacterial infection diagnoses concurrent with doubled viral RTI diagnoses and consistently by a major decrease in antibiotic prescription, without altering the overall mortality rate. Such a dramatic decrease in antibiotic consumption after ASP in a geriatric unit is rarely reported [20].

Interestingly, HO-CDI incidence in the GRU was directly correlated with the antibiotic consumption in the AGU throughout the study period: HO-CDI is linked to the antibiotic-induced dysbiosis of the intestinal microbiota. Consequently, patients receiving antibiotics during acute care (AGU) are at high risk of HO-CDI in the weeks following acute care, i.e., during their stay in GRU. Other studies have reported a strong association between HO-CDI and antibiotic consumption [3,4,5,6,7,8], and other ASPs have been successful in decreasing HO-CDI incidence in geriatric hospitals [3,9]. Despite the constant reduction in antibiotic consumption in the AGU, HO-CDI in the GRU increased again in 2018. We mainly assumed that antibiotic consumption in the GRU, rising in 2018, also influenced HO-CDI incidence in the GRU.

Several limits have to be acknowledged. First, this was an observational study without demonstration of any link of causality between ASP and antibiotic consumption or CDI incidence. Second, the different classes of antibiotics have not been analyzed separately, although their respective impacts on HO-CDI incidence largely differ [21,22]. Third, there was no evaluation whether this ASP was associated with a decrease in overall antibiotic resistance; however, such an association is particularly difficult to establish in clinical practice [20,23]. Fourth, a comprehensive multidisciplinary approach is required to fight against the HO-CDI epidemic efficiently, and ASP is only one of its components. Practices such as the hand hygiene of hospital staff, room cleaning, and contact isolation of patients with suspected infection have proven their efficiency [6,24]. Probiotic use has also been associated with a decreased incidence of HO-CDI [25,26]. However, there was no change in hygiene practices or probiotic prescriptions in our hospital during the study period.

## 5. Conclusions

In response to an HO-CDI outbreak in a geriatric hospital, an ASP centered on reducing the overdiagnosis of bacterial infections in the AGU was associated with an almost fifty percent drop in the number of diagnosed cases of bacterial infections, which was followed by a major decrease in antibiotic consumption and a correlated reduction in HO-CDI incidence.
